# Effects of calcium and magnesium on acute and chronic neurotoxicity caused by oxaliplatin: A meta-analysis

**DOI:** 10.3892/etm.2012.678

**Published:** 2012-08-22

**Authors:** RUI AO, YU-HUI WANG, RUI-WEN LI, ZHENG-RONG WANG

**Affiliations:** 1 Key Laboratory of Chronobiology of Health Ministry, Basic and Forensic School, Sichuan University;; 2 Department of Oncology, Sichuan Academy of Medical Sciences, Sichuan Provincial People’s Hospital, Chengdu, Sichuan 610041, P.R. China

**Keywords:** calcium and magnesium, neurotoxicity, oxaliplatin, chemotherapy

## Abstract

The primary toxicity of oxaliplatin is neurotoxicity. Calcium and magnesium (Ca/Mg) are reported to be beneficial in protecting against this adverse effect. However, the results obtained from clinical trials are not definitive. The aim of this study was to evaluate whether Ca/Mg alleviates the neurotoxicity of oxaliplatin by performing a meta-analysis of the literature involving available randomized controlled trials. Systematic searches for trials were undertaken from the Cochrane Library, MEDLINE, CENTRAL, Embase, CBMdisc and CNKI databases without language limitations. The primary outcome was severe chronic neurotoxicity and the secondary outcome was acute neurotoxicity. Four randomized double-blind trials met the search criteria. The odds ratio (OR) comparing Ca/Mg treatment with placebo was 0.44 (0.23–0.85, P=0.01) for severe chronic neurotoxicity of oxaliplatin (grade ≥2) and 0.41 (0.11–1.49, P=0.18) for acute neurotoxicity. In conclusion, Ca/Mg treatment does not reduce the incidence of acute neurotoxicity of oxaliplatin, but does reduce the incidence of severe chronic neurotoxicity (grade ≥2). No differences were observed in the outcomes of chemotherapy. Thus, Ca/Mg treatment is recommended for use as an adjunct with oxaliplatin.

## Introduction

Oxaliplatin is a third-generation platinum compound with structural differences from earlier platinates. It binds and cross-links with DNA, forming DNA adducts, thus inhibiting DNA replication and transcription. Chemotherapy regimens that include oxaliplatin are widely used in the treatment of colorectal cancer, having beneficial effects with response rates as high as 53% and a relatively low incidence of adverse effects, such as nausea, neutropenia, and vomiting (1,2). However, neurotoxicity restricts its use and causes patient discomfort. The neurotoxicity observed with oxaliplatin can manifest as one of two distinct syndromes: a transient, acute syndrome that can appear during or shortly after infusion of oxaliplatin and a dose-limiting, cumulative sensory neuropathy.

The acute neuropathy occurs within minutes of infusion of oxaliplatin and is able to remain for 1–2 days. Frequently, this presents as paresthesia, tingling or an unusual sensation in the tongue, jaw spasms or limb stiffness, and may be triggered or aggravated by exposure to cold. The incidence of acute neuropathy ranges from 81.5 to 98% (3). The cumulative, chronic neurotoxicity presents as paresthesia and proprioceptive changes that do not resolve between cycles of chemotherapy, and occurs in approximately 15% of patients after cumulative oxaliplatin doses of 780–850 mg/m^2^, leading to functional impairment in approximately 10% of patients (2). The mechanism of oxaliplatin-induced neurotoxicity remains unclear.

One of the metabolites of oxaliplatin, oxalate, is a calcium chelator that is thought to have a deleterious effect on specific neuronal sodium channels, including voltage-gated and calcium-dependent channels (4–6). Certain agents are administered in an attempt to prevent neurotoxicity, such as glutathione, carbamazepine, gabapentin, amifostine, celecoxib, and a combination of calcium and magnesium (Ca/Mg). Ca/Mg is relatively widely used (7–11). Ca/Mg is able to form a chelate with oxalate and reduce its effect on neuronal sodium channels. Intravenous calcium gluconate and magnesium sulfate are used at some institutions with the hopes of decreasing subsequent neuropathy (12–14). However, there has been some controversy about the effects of Ca/Mg on the reduction of neurotoxicity and whether it decreases the antitumor activity of oxaliplatin (15,16). The purpose of this study was to evaluate the effects of Ca/Mg on the efficacy of oxaliplatin, using a meta-analysis of randomized trials, by assessing whether Ca/Mg has the potential to reduce oxaliplatin-induced neurotoxicity.

## Materials and methods

### 

#### Selection criteria

Trials were eligible for inclusion based on the following criteria: i) patients received chemotherapy that included oxaliplatin; ii) trials were described as randomized clinical trials; iii) published trials included a treatment group receiving Ca/Mg during chemotherapy and a control group that did not receive Ca/Mg; and iv) the evaluation criteria of chronic neurotoxicity of oxaliplatin in the published trials adopted the neurotoxicity grade of the National Cancer Institute’s Common Toxicity Criteria (NCI-CTC) version 3.0, or included the acute neuropathy of oxaliplatin (Sanofi-Aventis: Eloxatin: Assessing neuropathy. Available at http://www.eloxatin.com/. Accessed February 11, 2010). Trials were excluded if they did not meet the criteria above and included the following: i) they involved animal or *in vitro* studies; ii) they did not represent primary research (e.g., review articles, letters to the editor); or iii) they represented duplicate publications of other studies previously identified in our systematic evaluation.

#### Search strategy

Retrieval of trials was performed through searches of MEDLINE (January 1966 to October 2011), Embase (January 1966 to October 2011), CBMdisk (Chinese Biomedical Database; January 1978 to October 2011), CNKI (Chinese National Knowledge Infrastructure; January 1979 to October 2011), and CENTRAL (the Cochrane Central Register of Controlled Trials; January 1966 to October 2011). The databases were searched without language limitations. The search was designed to initially find all trials involving the following terms: oxaliplatin or FOLFOX or FOLFOX4 or FOLFOX6 or XELOX and calcium or magnesium or Calcium gluconate or Ca/Mg. A manual search for general reviews on neurotoxicity of oxaliplatin and references from published clinical trials was conducted. The search results were downloaded to reference databases and screened further.

#### Outcome measurements

Outcome measurements of these trials included the following: i) the main endpoint was the proportion of severe, chronic neurotoxicity (grade ≥2); ii) the second endpoint was the extent of acute neuropathy; and iii) the third endpoint was the effects of chemotherapy. These data were extracted from actual numbers reported in the trials.

### Review methods

#### Data checking

Methodological quality of trials was evaluated according to the Jadad quality scores (17), which include secure method of randomization, allocation concealment, patient and observer blinding, and withdrawal. Based on these criteria, the studies were divided into a high-quality group (score ≥4) and a low-quality group (score <4). Two reviewers independently assessed the eligibility of each trial.

#### Data extraction

Each study included in the meta-analysis was read and the data were extracted and cross-checked independently by two reviewers, and discrepancies were resolved by discussion. The following information was extracted from each included trial: i) characteristics of the methods used (randomization procedure, allocation concealment, blinding procedure, withdrawal and reasons); ii) the number of patients allocated and patient characteristics; iii) the regimen of chemotherapy and the number of cycles patients received; and iv) the extent of acute and chronic neurotoxicity.

#### Statistical analysis

Patients were pooled for the meta-analysis by a biostatistician. Analysis was performed using RevMan 5.0. If P>0.10 or I^2^ ≤50%, heterogeneity of the trial was considered acceptable and differences between the OR and 95% CI were computed by the fixed-effects model. If P≤0.10 or I^2^ >50%, differences between the OR and 95% CI were computed by the random-effects model.

## Results

### 

#### Description of trials

A total of 135 studies were retrieved. Only five studies were eligible for our meta-analysis. One trial was excluded due to insufficient data, as only grade 3 chronic neurotoxicity was evaluated. The remaining four trials were described as randomized, doubled-blind studies. Two of the four trials evaluated the incidence of acute neurotoxicity caused by oxaliplatin, and chronic neurotoxicity was evaluated in all four trails according to the NCI-CTC criteria. Chronic neurotoxicity of grade ≥2 was considered as severe neurotoxicity. A total of 202 patients completed the evaluation for chronic neurotoxicity. Two of the four trials evaluated chronic neurotoxicity at the end of the sixth cycle of chemotherapy, and all patients were required to have completed at least two cycles of chemotherapy and were expected to complete 12 cycles or six months of chemotherapy. For chemotherapy outcome, one trial compared the treatment outcome between the two groups according to the response evaluation criteria in solid tumors (RECIST) (18), and one trial compared the risk of recurrence between the two groups whose patients underwent tumor resection. Characteristics of the included trials are listed in Table I. NCI-CTC criteria are listed in Table II.

#### Meta-analysis outcomes

**Figure 1:**
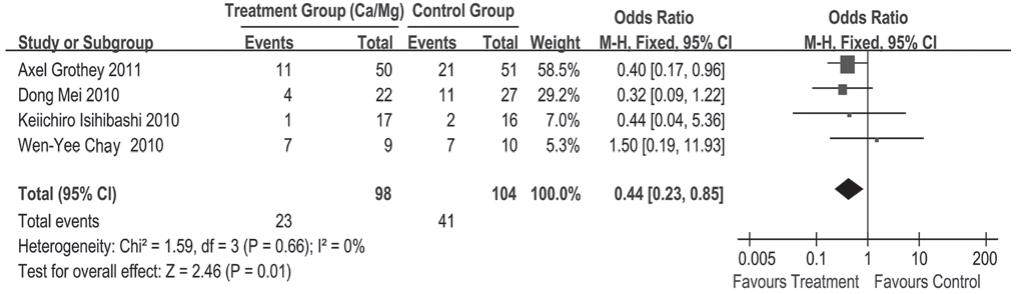
Meta-analysis of chronic neurotoxicity of oxaliplatin. Chemotherapy with calcium/magnesium (Ca/Mg) versus chemotherapy with placebo.

The four trials included in this study evaluated chronic neurotoxicity of oxaliplatin by NCI-CTC 3.0. The pooled analysis showed that compared with placebo, Ca/Mg significantly reduced the number of patients with a neurotoxicity grade of ≥2, with an OR of 0.44 (95% CI, 0.23–0.85). The fixed-effects model was used because heterogeneity of the results was acceptable (P=0.66, I^2^ = 0) (Fig. 1).

**Figure 2:**
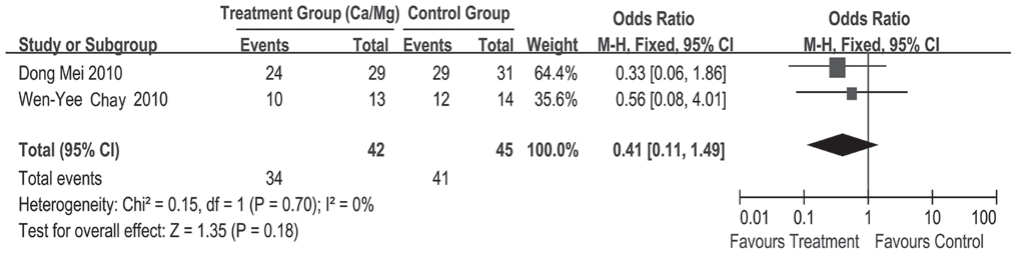
Meta-analysis of acute neurotoxicity of oxaliplatin. Chemotherapy with calcium/magnesium (Ca/Mg) versus chemotherapy with placebo.

We identified two trials containing evaluation of acute neurotoxicity of oxaliplatin. The fixed-effects model was used (P=0.70, I^2^, 0). Analysis showed that Ca/Mg had no effect on the acute neurotoxicity of oxaliplatin, with an OR of 0.41 (95% CI, 0.11–1.49) (Fig. 2).

#### Treatment outcomes of chemotherapy

The effects of chemotherapy on unresectable tumors in both the placebo and Ca/Mg groups were compared in one trial. No significant differences were observed in the response rates, disease control rates, or survival times between the two groups. The effects of adjuvant chemotherapy were observed in another trial (22), the results of which showed that the use of Ca/Mg did not seem to increase the recurrence of tumors.

## Discussion

The meta-analysis, which was based on data provided by four randomized trials, compared the administration of Ca/Mg with placebo regarding protection from the neurotoxicity of oxaliplatin. Ca/Mg did not show any marked benefit against acute neurotoxicity, although a significant effect on the reduction of severe chronic neurotoxicity caused by oxaliplatin was evident. Chemotherapy regimens including oxaliplatin are currently considered to be a standard of care in the management of adjuvant and metastatic colorectal cancers (1,23,24). However, the neurotoxicity that often occurs alters patient quality of life and may lead to postponement or even interruption of oxaliplatin therapy.

In 2004, Gamelin *et al* published a clinical trial concluding that calcium and magnesium infusion significantly reduced the incidence and severity of peripheral neuropathy secondary to oxaliplatin (13). Those authors administered 1 g of calcium gluconate and 1 g of magnesium sulfate as an infusion 1 or 2 h prior to oxaliplatin infusion and after the oxaliplatin infusion finished. In their retrospective cohort of 161 patients treated with oxaliplatin plus 5-fluorouracil/leucovorin for advanced colorectal cancer, the percentage of patients with grade 3 distal paresthesia was significantly lower in the Ca/Mg group (7 vs. 26%, P=0.001) (13).

In a retrospective study by Knijn *et al* (25), 755 previously untreated, advanced colorectal cancer patients were enrolled; the Ca/Mg^+^ group comprised 551 patients and the Ca/Mg^−^ group comprised 181 patients. Incidence of all grades of neurotoxicity in the Ca/Mg^+^ and Ca/Mg^−^ groups was 85 and 92%, respectively (P=0.02), and the incidence of neurotoxicity of grade ≥2 was 40 and 45%, respectively (P=0.22). The median progression-free survival (PFS) in the Ca/Mg^+^ versus Ca/Mg^−^ group was 10.1 versus 10.7 months (P=0.92), the median OS was 19.8 versus 20.7 months (P=0.10), and the response rate was 43.1 versus 50% (P=0.11). The results also demonstrate that Ca/Mg infusions significantly reduce the likelihood of all grades of neurotoxicity, but had no significant effect on the efficacy of treatment (25).

In contrast to the present results, the CONCEPT (Combined Oxalipatin Neurotoxicity Prevention Trial) study, a clinical trial of patients who were administered Ca/Mg infusions to reduce the neurotoxicity resulting from oxaliplatin treatment, was prematurely terminated in 2007 after it had been ongoing for 2 years. The independent data monitoring committee found that the Ca/Mg infusion reduced the efficacy of the chemotherapy regimen (14). Subsequently, Gamelin stated that Ca/Mg infusion has no impact on oxaliplatin efficacy (26). Thus, controversy exists regarding the value of Ca/Mg, focusing on whether Ca/Mg reduces the incidence of neurotoxicity or affects the treatment outcomes of oxaliplatin, and the concern that some of the prospective, randomized double-blinded trials did not show convincing evidence supporting the use of Ca/Mg.

On the basis of our meta-analysis of high-quality prospective trails, we suggest that Ca/Mg is able to reduce the incidence of severe chronic neurotoxicity of oxaliplatin without any evidence that it has an impact on efficacy. Accordingly, we suggest Ca/Mg infusions be continued in order to reduce oxaliplatin neurotoxicity.

## Figures and Tables

**Table I:** Basic characteristics of trials included in this study.

Trial (Refs.)	Country	Site of tumor	Enrolled patients		Chemotherapy regimen	Dose of Ca/Mg		Placebo
			Ca/Mg	Control		Ca	Mg	
Grothey *et al* 2011 (19)	USA	Colorectal	50	52	FOLFOX-4, FOLFOX-6	1 g	1 g	Identical appearance
Dong *et al* 2010 (20)	China	Gastrointestinal tract	29	31	FOLFOX-4	1 g	1 g	Normal saline l
Ishibashi *et al* 2010 (21)	Japan	Colorectal	17	16	mFOLFOX-6	0.85 g	0.72 g	5% glucose
Chay *et al* 2010 (22)	Singapore	Colorectal	13	14	XELOX, FOLFOX-4	1 g	1 g	Normal saline

FOLFOX-4, 2-h infusion of leucovorin (200 mg/m^2^/d) followed by a fluorouracil bolus (400 mg/m^2^/d) and 22-h infusion (600 mg/m^2^/d) for two consecutive days every 2 weeks, together with oxaliplatin 85 mg/m^2^ as a 2-h infusion on day 1; mFOLFOX-6: 2-h infusion of leucovorin (400 mg/m^2^) followed by a fluorouracil bolus (400 mg/m^2^) and 46-h infusion (2,400 mg/m^2^) every 2 weeks, together with oxaliplatin 85 mg/m^2^ as a 2-h infusion on day 1; XELOX, oral capecitabine 1000 mg/m^2^ twice a day for Days 1–14 and oxaliplatin 130 mg/m^2^ on Day 1 every 21 days; Ca/Mg, calcium gluconate and magnesium sulfate, which were used on the first day of chemotherapy.

**Table II:** National Cancer Institute common toxicity criteria (NCI-CTC).

Scale	NCI-CTC grading for neuropathy
Grade 1	Asymptomatic, loss of deep tendon, reflexes, or paresthesia (including tingling), but not interfering with function
Grade 2	Sensory alteration or paresthesia (including tingling) interfering with function, but not ADL
Grade 3	Sensory alteration or paresthesia interfering with ADL
Grade 4	Disabling

NCI-CTC, National Cancer Institute’s Common Toxicity Criteria. ADL, activities of daily living.

**Table III:** Quality assessment of trials included in this study.

Trial (Refs.)	Randomization	Allocation concealment	Blind	Withdrawal and dropout	Jadad score
Grothey *et al* 2011 (19)	Without details	Without details	Double-blind	Well reported	6
Dong *et al* 2010 (20)	Without details	Without details	Double-blind	Without details	4
Ishibashi *et al* 2010 (21)	Well reported	Appropriate	Double-blind	Well reported	7
Chay *et al* 2010 (22)	Without details	Without details	Without details	Well reported	4
